# CD99 triggering induces methuosis of Ewing sarcoma cells through IGF-1R/RAS/Rac1 signaling

**DOI:** 10.18632/oncotarget.13160

**Published:** 2016-11-07

**Authors:** Maria Cristina Manara, Mario Terracciano, Caterina Mancarella, Marika Sciandra, Clara Guerzoni, Michela Pasello, Andrea Grilli, Nicoletta Zini, Piero Picci, Mario P. Colombo, Andrea Morrione, Katia Scotlandi

**Affiliations:** ^1^ CRS Development of Biomolecular Therapies, Experimental Oncology Laboratory, Istituto Ortopedico Rizzoli, Bologna 40136, Italy; ^2^ PROMETEO Laboratory, STB, RIT Department, Istituto Ortopedico Rizzoli, Bologna 40136, Italy; ^3^ CNR, National Research Council of Italy, Institute of Molecular Genetics, Bologna 40136, Italy; ^4^ SC Laboratory of Musculoskeletal Cell Biology, Istituto Ortopedico Rizzoli, Bologna 40136, Italy; ^5^ Molecular Immunology Unit, Department of Experimental Oncology and Molecular Medicine, Fondazione IRCCS “Istituto Nazionale dei Tumori,” Milan 20133, Italy; ^6^ Department of Urology and Biology of Prostate Cancer Program, Sidney Kimmel Cancer Center, Thomas Jefferson University, Philadelphia, PA 19107, USA

**Keywords:** antibody, CD99, cell death, Ewing sarcoma, RAS

## Abstract

CD99 is a cell surface molecule that has emerged as a novel target for Ewing sarcoma (EWS), an aggressive pediatric bone cancer. This report provides the first evidence of methuosis in EWS, a non-apoptotic form of cell death induced by an antibody directed against the CD99 molecule. Upon mAb triggering, CD99 induces an IGF-1R/RAS/Rac1 complex, which is internalized into RAB5-positive endocytic vacuoles. This complex is then dissociated, with the IGF-1R recycling to the cell membrane while CD99 and RAS/Rac1 are sorted into immature LAMP-1-positive vacuoles, whose excessive accumulation provokes methuosis. This process, which is not detected in CD99-expressing normal mesenchymal cells, is inhibited by disruption of the IGF-1R signaling, whereas enhanced by IGF-1 stimulation. Induction of IGF-1R/RAS/Rac1 was also observed in the EWS xenografts that respond to anti-CD99 mAb, further supporting the role of the IGF/RAS/Rac1 axis in the hyperstimulation of macropinocytosis and selective death of EWS cells. Thus, we describe a vulnerability of EWS cells, including those resistant to standard chemotherapy, to a treatment with anti-CD99 mAb, which requires IGF-1R/RAS signaling but bypasses the need for their direct targeting. Overall, we propose CD99 targeting as new opportunity to treat EWS patients resistant to canonical apoptosis-inducing agents.

## INTRODUCTION

Ewing sarcoma (EWS) is a malignant mesenchymal tumor of children and young adults, with unmet clinical solution and relevant social impact. Despite the use of intensive multidrug treatments combined with surgery and/or radiotherapy, this highly invasive bone tumor forms lung and/or bone metastases in about 30-40% of patients with localized tumor, while approximately 30% of patients have detectable metastasis at diagnosis. Metastatic patients have very poor prognosis and treatment remains a challenge. The characterization of EWS genomes has poorly contributed to the identification of novel therapeutic strategies. EWS shows very low rate of somatic mutations [1, 2] confirming the dependence of this tumor on the oncogenic chimeric EWS–FLI1 protein [3]. However, as a transcription factor, EWS–FLI1 is a puzzling drug target [4], and current therapy of EWS still depends on conventional cytotoxic drugs with no alternative options for patients relapsing after first line therapies. This is relevant being EWS generally resistant to apoptotic cell death. Mechanisms of resistance rely on either alterations of the glutathione pathway [5], or the constitutive activation of the IGF-1R, TRKB, ErbB4 [6], caveolin-1 or PKCα [7], which may determine patients prognosis. The identification of novel mechanisms inducing cell death independently of canonical apoptosis is therefore imperative for developing new therapeutic approaches for EWS patients.

A potential method to kill cancer cells in a caspase-independent mechanism is the hyperstimulation of macropinocytosis, which can induce a form of non-apoptotic cell death, known as methuosis, death by macropinocytosis or catastrophic vacuolation [8–11]. This process is characterized by extreme accumulation of vacuoles in the cytoplasm, which compromises cell viability [12]. Methuosis was originally described after ectopic expression of the oncoprotein H-RAS in human glioblastoma, gastric carcinoma and osteosarcoma cells [13]. More recently, the demonstration that methuosis can be induced by different triggers (miRNAs, small molecules) in other tumors, such as papillary thyroid carcinoma [14], neuroblastoma [10], prostate cancer, breast, renal and lung cancer cells [15], has raised the possibility that agents capable of disrupting the physiological macropinosome trafficking pathways might be exploited for inducing cancer cell death.

CD99, a cell surface molecule [16] involved in several biological processes including migration, cell death and differentiation [17–19], is consistently highly expressed in EWS cells and is crucial for EWS malignancy. In this paper, we demonstrate that CD99 engagement by the 0662 anti-CD99 monoclonal antibody (mAb) [20, 21] induces cell death of EWS cells through a non-apoptotic pathway resembling methuosis, which requires the activation of insulin-like growth factor receptor 1 (IGF-1R) [22, 23] and RAS-Rac1 downstream signaling. These data define a novel role for CD99 and the IGF-1R/RAS pathway in methuosis and identify a novel targeting approach for EWS cells, including chemoresistant variants.

## RESULTS

### CD99 triggering by 0662mAb induces massive macropinocytosis that results in cell death

CD99 promotes cancer cell death when triggered by specific antibodies, such as 0662, O13 murine mAbs or the human single chain fragment variable diabody (dAbd C7) [20]. Engagement of CD99 with antibodies induced fast (within 15 min) and massive annexin V exposition to the outside layer of cell membrane, MDM2 ubiquitination and degradation, reactivation of p53 signaling and mitochondrial depolarization [20, 21]. All EWS cells express CD99 [24] and we found that 13 patient-derived EWS cell lines, including chemotherapy-resistant variants, are all sensitive to CD99-induced cell death (Supplementary Table S1). Sensitivity of EWS cells to anti-CD99 antibodies correlated with the status of p53 (p=0.048, Fisher's exact test).

**Figure 1:**
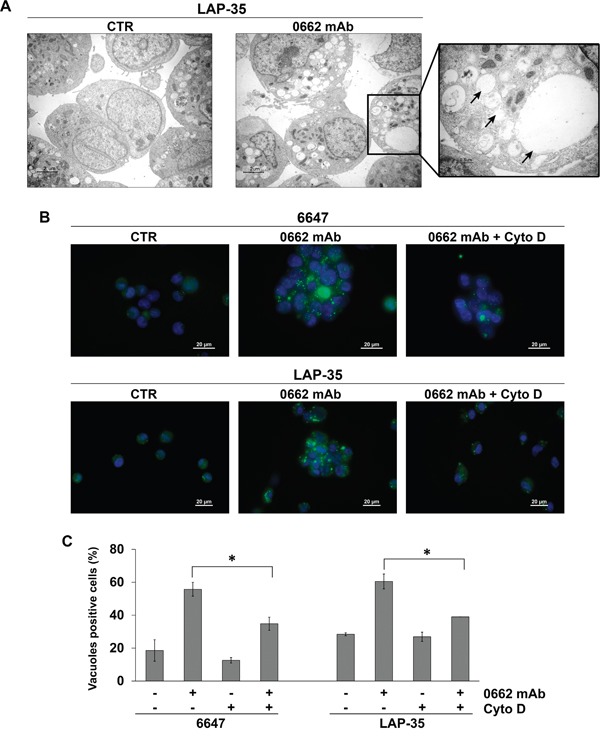
CD99 engagement by 0662 monoclonal antibody induces macropinocytosis in EWS cells **A.** Transmission electron microscopy of untreated (CTR) or 0662mAb-treated LAP-35 cells (scale bar 2μm). The right panel is a magnification of mAb-treated sample (scale bar 0.5μm). Arrows indicate empty vacuoles, void of cytoplasm and organelles. **B.** Lucifer yellow (LY) accumulation in 6647 or LAP-35 EWS cells in presence or not of Cytochalasin D (Cyto D) after 30 min 0662mAb exposure. Images were acquired with a Nikon ECLIPSE 90i with Plan Apo 60x/NA 1.4 DIC N2 (scale bar 20μm). **C.** Percentage of LY-positive 6647 and LAP-35 cells in presence or absence of 0662mAb (30 min) and/or Cyto D (60 min pretreatment). Results are represented as mean ± SEM of three independent experiments (*p<0.05, Student's *t* test).

However, loss of cell viability associated with CD99 engagement is not prevented by the caspase inhibitor z-VAD-fmk and dying cells lack features typical of canonical apoptosis (cell shrinkage, chromatin condensation, DNA fragmentation) [21]. Electron microscopy analysis showed that anti-CD99 mAb (3μg/ml, 30 min) induced massive vacuolization of EWS cells (Figure 1). The vacuoles observed in LAP-35 (Figure 1A) and 6647 (not shown) EWS cell lines were of various size (diameter range: 0.3-2.5μm), and characterized by a single membrane (Figure 1A: arrows), features consistent with macropinosomes [11]. Mitochondria and the double membrane typical of autophagosomes were rarely observed, suggesting that CD99-associated cell death might be related to methuosis [12], which is characterized by progressive accumulation of cytoplasmic vacuoles originating from macro- and/or micro-pinosomes [25]. Accordingly, necrostatin-1, an allosteric inhibitor of the death domain receptor-associated adaptor kinase RIP1 which is involved in necroptosis, did not protect LAP-35 cells from CD99-induced cell death (Supplementary Figure S1A). Similarly, treatments with either the autophagy inhibitor 3-methyladenine (3-MA), that prevents autophagosome formation, or knock-down of ATG7, a member of autophagic machinery, did not reduce CD99-mediated cytotoxicity (Supplementary Figure S1B and S1C), despite the accumulation of LC3-IIB in LAP-35 EWS cells (Supplementary Figure S1D) and a punctuate pattern of LC3 distribution as detected by immunofluorescence analysis (Supplementary Figure S1E). These results indicate that autophagy, even if induced, is likely a compensatory stress response to anti-CD99 antibody treatment rather than a cell death mechanism.

Methuosis relies on the initial formation of vacuoles through clathrin-independent endocytosis and the progressive accumulation and enlargement of vacuoles up to the point of cytoplasmic membrane disruption [12]. Gene expression profile of 6647 EWS cells treated with anti-CD99 0662mAb suggests disruptions in endocytic processes (Supplementary Table S2). Enrichment analysis using Kyoto Encyclopedia of Genes and Genomes (KEGG) annotations revealed that the majority of positively modulated genes were related to endocytosis/lysosomal pathways, particularly after 60 and 120 min of treatments (Supplementary Table S2). Induction of endocytic-like processes by anti-CD99 mAb was confirmed by a rapid (30 min) incorporation of tracer lucifer yellow (LY), a hallmark of macropinosomes, in 6647 and LAP-35 cells after engagement of CD99 by 0662mAb (Figure 1B and 1C), while internalization of LY was very modest in untreated cells. The phenotype was prevented by the macropinocytosis inhibitor cytochalasin D (Figure 1B and 1C).

**Figure 2:**
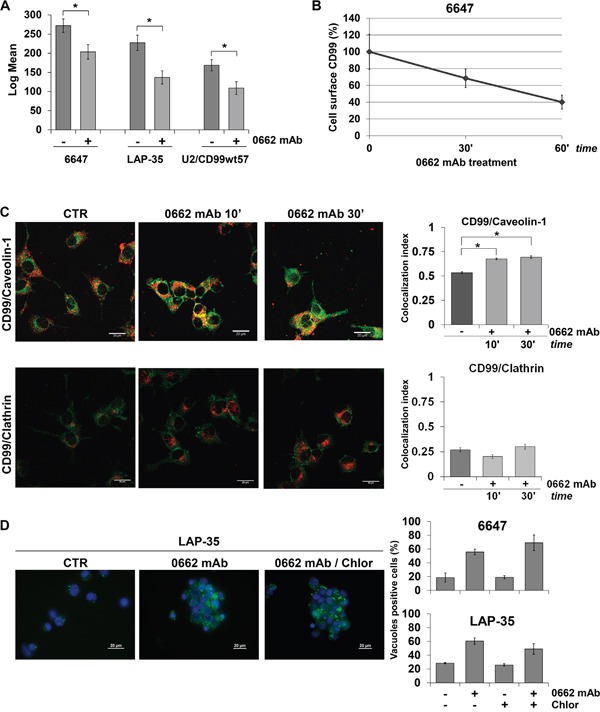
CD99 is internalized after 0662mAb exposure **A.** Intensity of CD99 surface expression before (−) and after (+) 0662mAb treatment (60 min) by flow cytometry. Results are represented as mean ± SEM of three independent experiments (*p<0.05, Student's *t* test). **B.** CD99 surface expression in 6647 cells by ELISA assay after 0662mAb treatment. Optical density was measured at 405nm. Data indicate mean relative CD99 expression ± SEM, referred to control absorbance. **C.** Colocalization between CD99 and Caveolin-1 or CD99 and Clathrin in LAP-35 cells before (CTR) and after 0662mAb treatments is shown by confocal microscopy. CD99 was labeled in green, Caveolin-1 or Clathrin in red (scale bar 20μm) (*p<0.05, Student's *t* test). Colocalization analysis was calculated by Nis Elements AR4.20.01 software (Nikon) and MCC was represented by histograms, as mean ± SEM of an average of one hundred cells from at least 10 independent fields. **D.** LY uptake in EWS cells exposed to 0662mAb for 30 min in presence or not of Chlorpromazine (10μg/ml) (scale bar 20μm). Left panels: representative images of LAP-35 cells. Right panels: percentage of LY-positive 6647 or LAP-35 cells in presence or absence of 0662mAb and/or Chlorpromazine. Results are represented as mean ± SEM of three independent experiments (Student's *t* test: n.s).

As EWS cells are not the best model to study endocytosis, having large nuclei, small cytoplasm and growing in suspension, we alternatively used U-2 OS osteosarcoma cells transfected with CD99 [26]. Treatment of EWS or osteosarcoma U2/CD99wt57 cells with 0662mAb triggered CD99 internalization, as shown by a significant decrease of CD99 cell surface levels measured by both flow cytometry (Figure 2A) and ELISA assay (Figure 2B).

CD99 significantly colocalized with caveolin-1, a major component of caveolae, while very limited colocalization was observed between CD99 and clathrin in both LAP-35 and U2/CD99wt57 cells (Figure 2C; Supplementary Figure S2A). Inhibition of clathrin-dependent pathways with chlorpromazine did not prevent LY uptake after treatment with anti-CD99 0662mAb (Figure 2D), indicating that CD99-induced vacuoles derive from macropinocytosis occurring preferentially through a caveolin-1-enriched, but clathrin-independent pathway.

CD99-induced macropinocytic vacuoles contain the small GTPase RAB5, a marker of early endosomes, as well as the late endosomal/lysosomal marker LAMP-1 (Supplementary Figure S2B). In fact, CD99 colocalized with both RAB5 and LAMP-1. Colocalization between CD99 and RAB5 was observed after 30-60 min, while colocalization with LAMP-1 was observed after 3h of antibody treatment and sustained up to 6h (Supplementary Figure S2B). Thus, CD99 triggering activates an endocytic pathway, which induces CD99 sorting into lysosomes-like structures. However, as described for methousis [12], CD99-induced vacuoles are not sufficiently acidic to sequester lysosomotropic dyes (not shown) or to allow acridine orange emission to change to orange/yellow (Supplementary Figure S2C), thus indicating that they are not fully functional lysosomes. Instead, these structures progressively accumulate in the cytoplasm, thereby compromising cell viability.

### CD99-induced methuosis requires activation of IGF-1R and RAS-Rac1 signaling

**Figure 3:**
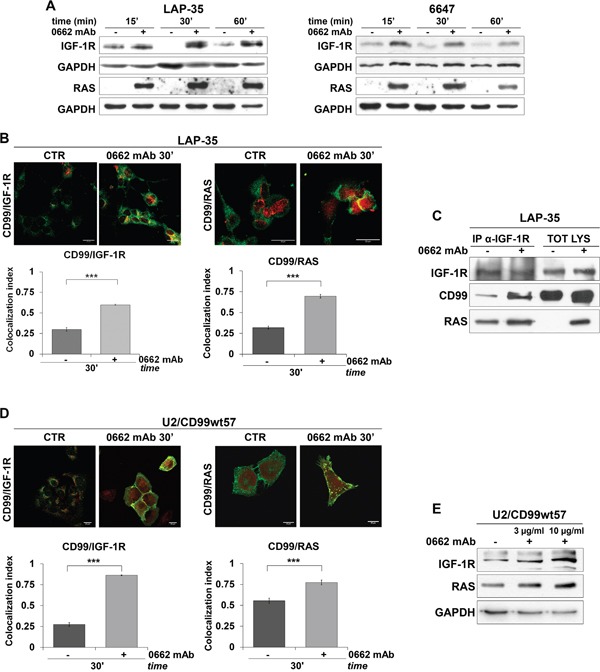
CD99, IGF-1R and RAS interact upon 0662mAb exposure **A.** Western blot analysis of IGF-1R and pan-RAS on cell lysates from control (−) or 0662mAb-treated (+) EWS cells at the indicated times. Equal sample loading was monitored by anti-GAPDH blotting. **B.** Confocal microscopy shows colocalization between endogenous CD99 and IGF-1R (left panel) or CD99 and RAS (right panel) in LAP-35 before (CTR) and after treatment with 0662mAb. Representative images are shown. CD99 was labeled in green, IGF-1R or RAS in red (scale bar 20μm). Values are expressed as mean ± SEM (***p<0.001, Student's *t* test). **C.** Coimmunoprecipitation of endogenous IGF-1R, RAS and CD99 in LAP-35 before and after treatment with 0662mAb. **D.** Confocal microscopy shows colocalization between endogenous CD99 and IGF-1R (left panel) or CD99 and RAS (right panel) in U2/CD99wt57 cells before and after treatment with 0662mAb. CD99 was labeled in green, IGF-1R or RAS in red (scale bar 20μm) (***p<0.001, Student's *t* test). **E.** Western blot analysis of IGF-1R and pan-RAS on cell lysates from control (−) or 0662mAb-treated (+) U2/CD99wt57 cells. Loading was monitored by anti-GAPDH blotting. Colocalization analysis was calculated by Nis Elements AR4.20.01 software (Nikon) and MCC was represented by histograms as mean values ± SEM (***p<0.001, Student's *t* test).

We previously reported a marked and rapid decrease of MDM2 protein levels following CD99 engagement [20]. MDM2 is an E3 ubiquitin ligase that antagonizes the tumor suppressor p53 [27]. In addition, MDM2 serves as an ubiquitin ligase for the IGF-1R, thereby promoting receptor degradation [28]. Thus, CD99 engagement, by altering MDM2 stability, may affect IGF-1R action and signaling. Accordingly, we detected induction of the IGF-1R and its downstream effector RAS following CD99 engagement (Figure 3A). Increased levels of IGF-1R are likely due to reduced degradation mediated by MDM2. In fact, engagement of CD99 did not alter transcription of the IGF-1R (Supplementary Figure S3A). In contrast, EWS cells stably or transiently overexpressing MDM2 showed reduced levels of IGF-1R in basal condition and upon CD99 engagement (Supplementary Figure S3B and S3C), confirming that the modulation of IGF-1R levels occurs through stabilization of the protein. In addition, the IGF-1R and RAS proteins colocalized as demonstrated by confocal microscopy (Figure 3B) and coimmunoprecipitated with CD99 in LAP-35 cells (Figure 3C). Similar results were recapitulated in U2/CD99wt57 cells (Figure 3D and 3E).

**Figure 4:**
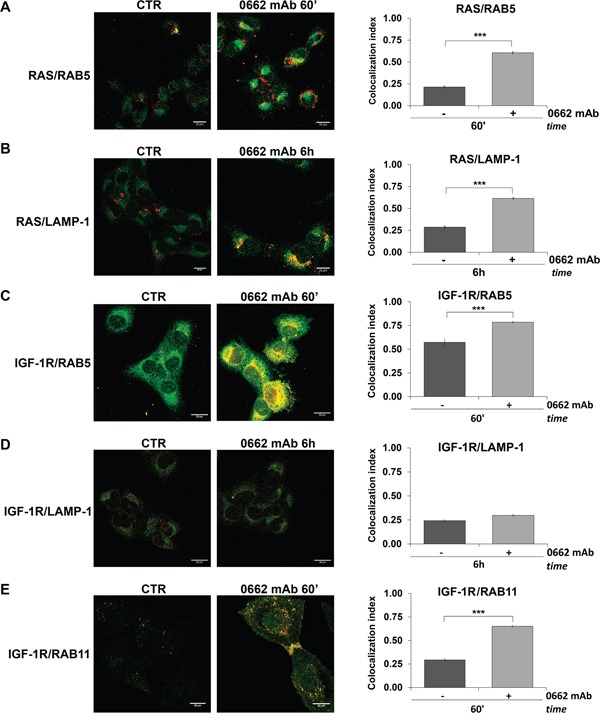
RAS and IGF-1R are sorted into early endosomes but the latter is recycled to the cell surface Confocal microscopy in U2/CD99wt57 before (CTR) and after 0662mAb treatment shows colocalization for: **A.** RAS (green) and RAB5 (red); **B.** RAS (green) and LAMP-1 (red); **C.** IGF-1R (green) and RAB5 (red); **D.** IGF-1R (green) and LAMP-1 (red); **E.** IGF-1R (red) and RAB11 (green) (scale bar 20μm). Representative images are displayed. Colocalization analysis was calculated by Nis Elements AR4.20.01 software (Nikon) and MCC was represented by histograms as mean values ± SEM. (***p<0.001, Student's *t* test).

Activated forms of RAS and the IGF-1R play an important role in regulating caspase-independent cell death, associated with cytoplasmic vacuolization [11, 13, 29]. In our cellular models, RAS and the IGF-1R were both internalized and sorted into early endosomes, as indicated by colocalization with RAB5 (Figure 4A and 4C), but while RAS followed CD99 and colocalized with LAMP-1 in late endosomes/lysosome-like vacuoles (Figure 4B), the IGF-1R did not (Figure 4D; Mander's overlap < 0.6) and colocalized instead with RAB11 (Figure 4E), a marker of the recycling compartment [30].

Accordingly, triggering of CD99 by 0662mAb in presence of cycloheximide, an inhibitor of protein synthesis, induced partial degradation of CD99 and RAS but not IGF-1R down-regulation (data not shown). Thus, in contrast to CD99 and RAS, which are partially down-regulated upon antibody treatment, the IGF-1R is likely recycled to the cell surface in RAB11-enriched compartments.

**Figure 5:**
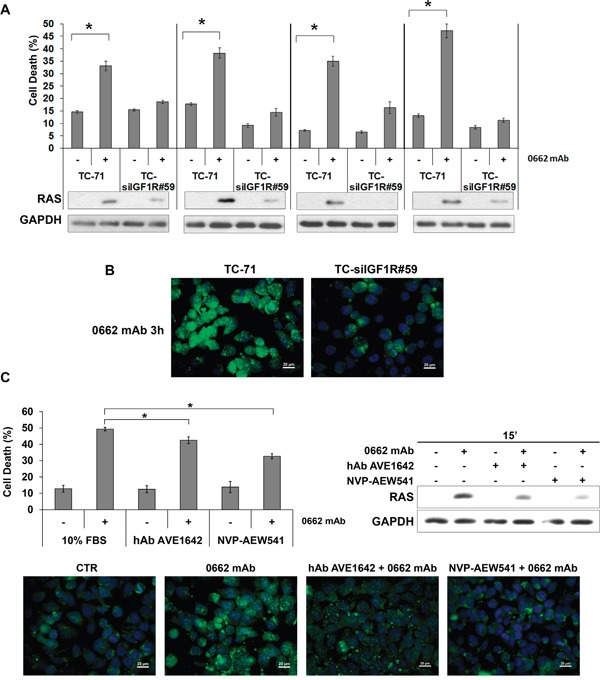
IGF-1R expression and activity are required for CD99-mediated methuosis **A.** CD99-induced cell death in parental or IGF-1R silenced TC-71 cells (Annexin V/PI) (upper panel). Results are represented as mean ± SEM of three independent experiments (*p<0.05, Student's *t* test). Western blotting evaluation of pan-RAS levels before (−) and after (+) treatment with 0662mAb in corresponding cells (lower panel). **B.** Vacuolization was assessed by Acridine Orange staining (AO) after 0662mAb treatment in parental (TC-71) or IGF-1R silenced cells (TC-siIGF1R#59) (scale bar 20μm). **C.** Experiments were performed in TC-71 cells before (−) and after (+) 15 min treatment with 0662mAb alone or in presence of either hAb AVE1642 or NVP-AEW541 (Annexin V/PI). Histograms (upper left panel) represent mean percentage ± SEM of dead cells (*p<0.05, Student's *t* test); western blotting (upper right panel) evaluates pan-RAS levels in the same conditions. Lower panels display AO staining before (CTR) and after 3h treatment with 0662mAb alone or in presence of either hAb AVE1642 or NVP-AEW541. For AO staining, cells were acquired using the microscope Nikon ECLIPSE 90i with Plan Fluor 40x/0.75 DIC M/N2. Pictures provided in the figures are all merged images.

In TC-71 cells silenced for IGF-1R expression (Supplementary Figure S4A), treatment with anti-CD99 0662mAb failed to induce cell death, (Figure 5A, Supplementary Figure S4B), RAS up-regulation (Figure 5A, Supplementary Figure S4C) and massive vacuolization significantly induced in TC-71 parental cells (Figure 5B, Supplementary Figure S4D). Conversely, inhibiting IGF-1R action in TC-71 EWS cells by either anti-IGF-1R neutralizing human antibody (hAb) AVE1642 [31] or the tyrosine-kinase inhibitor NVP-AEW541 [32] reduced CD99-induced cell death, RAS up-regulation and inhibited massive vacuolization (Figure 5C), indicating that CD99-mediated cell death requires a functional IGF-1R. The effect of the tyrosine kinase inhibitor NVP-AEW541 was stronger than that one observed after AVE1642 treatment, further suggesting that the IGF-1R activation likely contributes to anti-CD99 mAb activity. Accordingly, IGF-1 stimulation (50ng/ml, 15 min) of TC-71 EWS cells significantly (p-value=0.0226) increased the percentage of dead cells, promoted RAS up-regulation and led to massive vacuolization (Supplementary Figure S4E, S4F and S4G). Overall, these results indicate that the IGF-1R is necessary for CD99-driven RAS induction and cell vacuolization. In glioblastoma RAS-induced cytoplasmic vacuolization does not require the activation of MAPK or PI3K pathways [33]. Accordingly, the MEK inhibitor PD98059 or the Akt inhibitor LY294002, as well as wortmannin, did not block the induction of cell death in our EWS cellular models [21]. In addition, the suppression of ERK phosphorylation in 6647 and LAP-35 EWS cells by stable expression of h-RAS N17 (Supplementary Figure S5A), a dominant negative form of RAS that inhibits MAPK/ERK but not Rac1/JNK responses [34], did not affect but rather increased vacuolization (Supplementary Figure S5B) and cell death (Supplementary Figure S5C) upon CD99 triggering.

**Figure 6:**
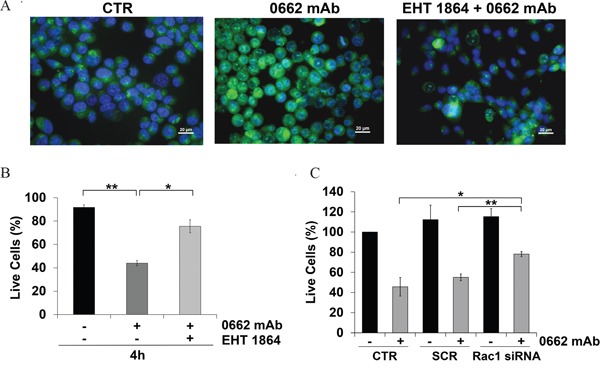
Rac1 inhibition reverts CD99-mediated effects **A.** AO staining was performed in 6647 cells before (CTR) or after exposure with 0662mAb (3h) alone or combined with the Rac1 inhibitor EHT 1864 (25 μM). For AO staining, cells were acquired using the microscope Nikon ECLIPSE 90i with Plan Fluor 40x/0.75 DIC M/N2. Pictures provided are all merged images (scale bar 20μm). **B**. Percentage of 6647 live cells before (−) and after (+) treatment with anti-CD99 0662mAb alone, or with Rac1 inhibitor EHT 1864 (25 μM for 48 h pretreatment) (trypan blue dye exclusion). **C**. Percentage of live 6647 cells before (−) and after (+) treatment with anti-CD99 0662mAb and/or with siRNA against Rac1. Cells were transfected by a scrambled (SCR) as control (trypan blue dye exclusion). Results are expressed as mean ± SEM of three or more independent experiments (Student's *t* test: *p< 0.05; **p<0.01).

**Figure 7:**
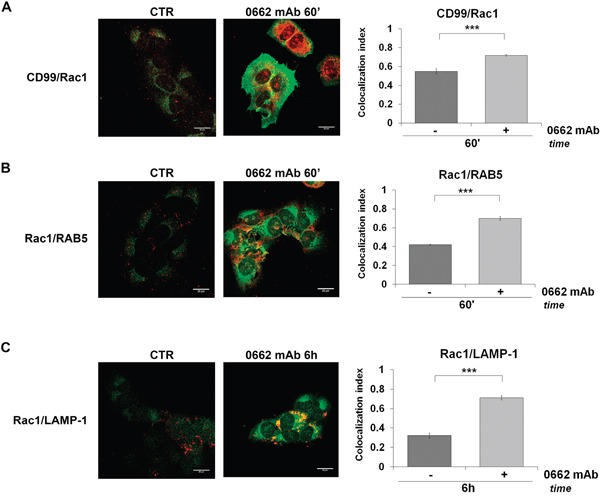
Rac1 colocalizes with CD99 and is sorted into vacuoles after 0662mAb treatment In U2/CD99wt57 before (CTR) and after 0662mAb treatment confocal microscopy shows colocalizations for **A.** CD99 (green) and Rac1 (red); **B.** Rac1 (green) and RAB5 (red); **C.** Rac1 (green) and LAMP-1 (red) (scale bar 20μm). Representative images are displayed. Colocalization index (MCC) represented by histograms was calculated by Nis Elements AR4.20.01 software (Nikon) and expressed as mean values ± SEM (***p<0.001, Student's *t* test).

**Figure 8:**
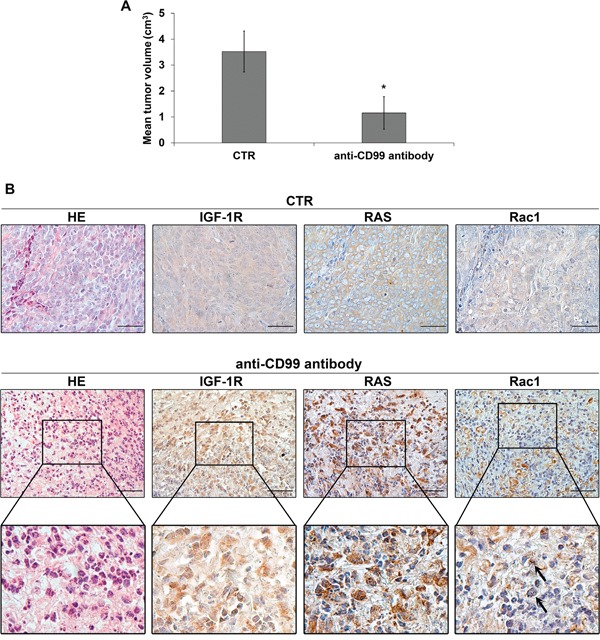
IGF-1R/RAS/Rac1 signaling is activated upon anti-CD99 treatment *in vivo* **A.** Mean tumor volumes in control and in CD99-antibody treated mice (Values are mean ± SEM, *p<0.05, Student's *t* test). **B.** Representative immunohistochemical evaluation of IGF-1R, RAS and Rac1 expression in untreated or anti-CD99 treated mice. Hematoxilin and Eosin staining was used to evaluate cell morphology. Enlarged views of the boxed regions are shown below. Arrows indicate Rac1 positive vesicles (scale bar 50μm).

On the contrary, no induction of vacuolization (Figure 6A) and a significant reversion of CD99-induced cell death was observed in EWS cells when we used either the specific Rac1 inhibitor EHT 1864 [35] (Figure 6B) or when endogenous Rac1 was depleted by siRNA approaches (Figure 6C). These results strongly indicate that the CD99/IGF-1R/RAS-mediated induction of methuosis in EWS occurs independently of MAPK/ERKs signaling but through Rac1 protein, a regulator of actin polymerization and a positive regulator of macropinocitosis and phagocytosis [36, 37]. Accordingly, Rac1 colocalized with CD99, RAB5 and LAMP-1 in vacuoles following the same kinetics previously observed for RAS (Figure 7A, 7B and 7C). Up-regulation of IGF-1R, RAS and Rac1 was also clearly detectable in 6647 xenografts after treatment with anti-CD99 mAb [20, 38] and Rac1-positive vesicles can be identified in responsive tumors (Figure 8A and 8B, arrows).

### CD99-induced methuosis is selective for cancer cells highly expressing CD99

**Figure 9:**
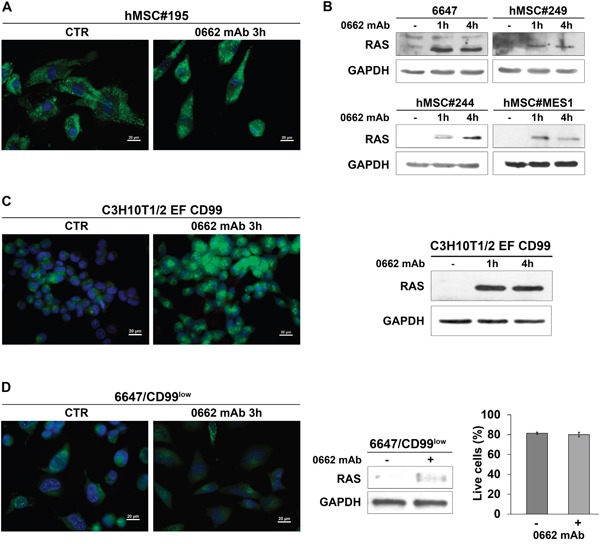
RAS and CD99 levels are crucial for 0662mAb-induced vacuolization **A.** AO staining of human mesenchymal stem cells (hMSCs) treated or not (CTR) with the anti-CD99 0662mAb. Representative merged images are shown (scale bar 20μm). **B.** Western blotting evaluation of pan-RAS levels before (−) and after treatment with anti-CD99 0662mAb in 6647 cells or hMSC cells. GAPDH was used as loading control. **C.** Left: AO staining of murine MSCs transfected with EWS/FLI1 and CD99 (C3H10T1/2 EF CD99) treated or not (CTR) with the anti-CD99 0662mAb. Representative merged images are shown (scale bar 20μm). Right: western blotting evaluation of pan-RAS levels before (−) and after treatment with 0662mAb in C3H10T1/2 EF CD99. GAPDH was used as loading control. **D.** Left: AO staining of 6647/CD99^low^ cells treated or not (CTR) with the anti-CD99 0662mAb. Representative merged images are shown (scale bar 20μm). Middle: western blotting evaluation of pan-RAS levels before (−) and after (+) treatment with 0662mAb for 1h in 6647/CD99^low^. GAPDH was used as loading control. Right: percentage of live cells before (−) and after (+) treatment with anti-0662mAb in 6647/CD99^low^ (Annexin V/PI assay). Values represent mean ± SEM of three independent experiments (Student's *t* test: p> 0.05).

We have previously demonstrated that engagement of CD99 does not affect viability of human hematopoietic and mesenchymal stem cells, constitutively expressing high levels of CD99 [20, 38]. Here, we showed that engagement of CD99 in mesenchymal stem cells did not induce macropinocytosis and massive vacuolization (Figure 9A) and cells showed minimal increase in RAS expression after CD99 triggering (Figure 9B).

On the contrary, when the murine C3H10T1/2 mesenchymal cells transfected with both EWS-FLI1 and CD99 were treated with the 0662mAb, they underwent catastrophic vacuolization, restored RAS induction (Figure 9C) and died [20]. Conversely, EWS cells resistant to the anti-CD99 0662mAb because of decreased CD99 levels on cell surface (6647/CD99^low^) did not induce RAS up-regulation and cytoplasmic vacuolization, when exposed to the antibody (Figure 9D). These data indicate that aberrant high expression of CD99 is absolutely required to activate catastrophic vacuolization and death.

These results additionally support the validity and specificity of anti-CD99 antibodies as a therapeutic tool for EWS treatment.

## DISCUSSION

In this paper, we provide the novel observation that engagement of CD99 with the 0662mAb activated a methuosis-like process that depends on the IGF-1R/RAS/Rac1 signaling cascade and lead to cell death in 13 patient-derived EWS cell lines, including those resistant to conventional chemotherapy. We also provided evidence that this mechanism is functional in 6647 EWS xenografts derived from mice treated with anti-CD99 mAb, supporting the *in vivo* relevance of our study. CD99 is expressed on the cell surface and it is detectable in virtually all EWS cases [24]. CD99 is critical for the pathogenesis of this tumor and it can be targeted in combination with conventional treatments [20, 38]. Thus, CD99 targeting has great clinical potential for EWS therapy.

CD99 triggering by 0662mAb mimics apoptotic stimuli [20, 21] but does not induce classical caspase-dependent apoptosis and triggers caveolin-regulated but clathrin-independent endocytosis, which accumulates defective vacuoles in the cytoplasm, thereby compromising viability. This process, known as methousis or death by macropinocytosis, has been previously described as an efficient mean of inducing cell death in different types of cancers [8–12]. In contrast to physiological macropinocytosis, methuosis is characterized by dysregulated clearance of vacuoles associated with insufficient recycling or fusion with other endosomal compartments. The nascent vacuoles acquire late endosomal/lysosomal markers such as LAMP-1 but do not contain lysosomal enzymes and acidic pH, as is the case of functional lysosomes [39]. Loss of viability may derive from cytoplasmic hyper-vacuolization associated with loss of metabolic capacity (decrease in ATP and mitochondrial potential) and plasma membrane integrity, without cell shrinkage and nuclear fragmentation associated with canonical apoptosis.

The signaling pathways that sustain methuosis are in part cell system-dependent but few common features have been so far defined. The process requires clathrin-independent endocytosis and mechanisms that converge on the regulation of actin dynamics and RAS signaling.

Maltese and colleagues firstly provided the evidence that induction of methuosis might only occur when the level of active RAS reaches a threshold sufficient to stimulate Rac1, thereby leading to inactivation of Arf6, a small GTPase that promotes recycling from clathrin-independent endosomes to plasma membranes [13]. Thus, Rac1-regulated vacuoles cannot recycle to the surface or fuse to lysosomes; instead, they fuse to each other and late endosomes creating large vacuoles that accumulate in the cytoplasm. The induction of methuosis caused by overexpression of RAS (G12V) is a slow process and may take several days. On the contrary, the anti-CD99 mAb as well as small molecules that drive methuosis [8, 40] can induce vacuolization within 1-4 hours, supporting their possible application in the clinic. Intriguingly, the cell-death promoting role of RAS has been characterized in the context of wild-type RAS up-regulation, whereas in cells carrying RAS mutations macropinocytosis was described as a mechanism supplying cancer cells with aminoacids to sustain proliferation [41]. This might indicate a profound difference between tumors with rare or high frequency of RAS mutations, highlighting the importance of the cellular context in defining therapeutic approaches. Significantly, EWS belongs to the class of tumors with low rate of RAS mutations [2, 42] suggesting that this particular tumor context may favor RAS signaling and methuosis.

Here, we elucidated the molecular events that, upon CD99 engagement, led to cell death and demonstrated that CD99, when engaged by 0662mAb, rapidly evokes caveolin-1-dependent endocytosis and fosters up-regulation of IGF-1R and RAS/Rac1 induction. Increased IGF-1R levels likely occurs through CD99-induced degradation of MDM2 [20], which impairs IGF-1R ubiquitination and degradation, as in fact MDM2, in addition to control of p53 levels, also serves as an ubiquitin ligase for the IGF-1R [43]. CD99 colocalizes with caveolin-1 [26], the IGF-1R, RAS and Rac1 in endosomes that are initially characterized by RAB5 expression and are later enriched in LAMP-1. However, while CD99, RAS and Rac1 are then sorted into late-endosomes that may partially retain the ability to merge with lysosomes, thereby inducing partial degradation of the molecules, the IGF-1R is rapidly sorted back to the cell surface through RAB11-dependent recycling. Thus, IGF-1R recycling may constitute an additional mechanism contributing to enhanced IGF-1R levels.

Stimulation of the IGF-1R was functionally necessary for CD99-induced RAS up-regulation and cell vacuolization. Considering that EWS cells display autocrine activation of IGF-1R, its recycling to cell membrane and consequent phosphorylation may work to ensure the maintenance of sustained levels of RAS and Rac1 signaling.

Inhibiting Rac1 interaction with its downstream effectors by either specific inhibitors [35] or siRNA approaches significantly inhibited CD99-induced vacuolization and cell death, indicating that Rac1 induced expression downstream of the IGF-1R/RAS complex is a critical determinant of CD99-induced cell death. At least two additional groups have identified small molecules that specifically induce methuosis by mechanisms dependent on Rac1 activity [8, 40]. Our work extends these observations showing the up-regulation of IGF-1R/RAS/Rac1 signaling after CD99 triggering *in vivo* and the formation of Rac1-positive vesicles in responsive tumors.

The discovery that anti-CD99 0662mAb exerts its anti-tumor action by enhancing IGF-1R/RAS/Rac1 activity may seem paradoxical. In fact, besides the well-known role of IGF-1R in promoting EWS cell malignancy [22, 44], Rac1 has been discovered as a key mediator of EWS cell invasion and metastasis [45, 46]. However, we believe that the induction of methuosis may explain this paradox. Rapid stimulation of physiological macropinocytosis upon IGF-1 exposure is one of the early events of IGF-1R signaling [47] and occurs to sustain cell proliferation and survival. The interaction between the IGF-1R and CD99, triggered by the 0662mAb, may modify actin dynamics and shift physiological macropinocytosis towards methuosis. Neither RAS nor Rac1 are mutated in EWS [2, 42], where aberrant activity is the result of altered expression and localization [11, 48]. Thus, CD99 may affect Rac1 downstream molecular interactions and specificity of response by sequestering RAS-Rac1 into vacuoles.

The results presented in this paper may have important clinical implications. Our data suggest that combining IGF-1R inhibitors with anti-CD99 mAb may have an antagonistic effect. In contrast, CD99 triggering exploits the constitutive activation of the IGF-1R, which is sustained by EWS-FLI [49], and drives cells toward death.

Toxicity remains a major issue in the drug development process and it is particular relevant for pediatric diseases. Here we showed that treatment with anti-CD99 0662mAb of normal human bone-marrow derived MSC cells, expressing CD99 at levels similar to EWS cells [20], did not induce hyperstimulation of vacuoles or RAS up-regulation. Accordingly, MSC cells are not susceptible to cell death, confirming previous results in other cellular models [8, 11, 15, 41, 50]. When MSC cells ectopically expressed EWS-FLI and CD99, they regain the ability to respond to anti-CD99 antibody entering a methuosis-like process. The effects are more dramatic in malignant cells with higher expression of CD99, as shown in CD99-resistant EWS cells. In addition, reactivation of p53 following CD99 engagement [20] may synergize with the death-promoting effects of RAS [51], further increasing treatment efficacy. Both p53 reactivation [20] and RAS induction could not be triggered in normal MSC cells after treatment with anti-CD99.

Overall, our data prove that CD99-induced macropinocytosis selectively destroys EWS cells, even when they are chemoresistant. As of today, antibodies against CD99 and few other small molecules are the only agents that may specifically induce methuosis [8, 40]. CD99 triggering activates an “apoptotic mimicry” mechanism that induces a methuosis-like process leading to cell death. We clearly show that CD99-antibody exploits a constitutively active IGF-1R to initiate RAS/Rac1 signaling, defective vacuolization and death. Interestingly, the process is enhanced in the presence of high concentration of IGF-1, which leads to increased percentage of EWS cell death. This is critical considering that IGFs are the prevalent growth factors of bone. Their release by tumor cell-induced bone destruction may therefore activate an autocrine mechanism, which may improve treatment efficacy against primary and metastatic tumors.

## MATERIALS AND METHODS

### Cell lines and primary cell cultures

EWS cell lines were grown as previously described [20, 26]. 6647 and TC-71 were kindly provided by T.J. Triche (Children's Hospital, Los Angeles, CA, 1994); RD-ES, SK-ES-1, SK-N-MC (1994) were provided by American Type Culture Collection, ATCC (Rockville, MD); H825 and H-1474-P2 were kindly provided by Prof Llombart-Bosch (2001); C3H10T1/2 EF CD99 was kindly provided by F. Lecanda, (2007) [52]. LAP-35 (1987); IOR/BRZ_2010 (1995); U2/CD99wt57 (2001) [26]; TC-71 siIGF-1R (2006); TC/ET 12nM (2002) [53]; 6647/CD99^low^ (2009) [52] were previously established in our laboratory; DOXO variants of TC-71 was obtained by transfection with a vector containing *MDR1* cDNA and selected in doxorubicin (30ng/ml, SIGMA) [53]. LAP-35 variants stably overexpressing MDM2 were previously characterized [20]. All cell lines were tested for mycoplasma (MycoAlert mycoplasma detection kit, Lonza) and authenticated by STR PCR analysis within last year (GenePrint^®^ 10 System or POWERPLEX EXS 17 fastsystem, Promega). Bone marrow-derived human mesenchymal (hMSC) stem cells were obtained from healthy donors or patients with benign bone lesions. hMSC cells characterized for mesenchymal markers and differentiative ability were used at early passages.

### Stable and transient transfections and lentiviral infections

Transfections were performed using Calcium Phosphate transfection Kit or Lipofectamine 2000 (Life Technologies) according to the manufacturer's protocol. Transient transfection of MDM2 gene was performed with pCMV-MDM2 [20]. Transient silencing of Rac1 was obtained by using anti-Rac1 oligos as previously described [54]. For stable silencing of IGF-1R, TC-71 cells were transfected with pSilencer™ −2.1.U6 neo (Ambion) containing the sequence for IGF-1R short hairpin RNA and selected with G418 (500 μg/ml). 6647 and LAP-35 cells were infected with pBABEpuro-HRASN17 or pBABEpuro empty vector supernatants, and DN cells were selected with puromycin (500 ng/ml). pLKO.1 shAtg7-E8 (TET-ON inducible) and pGIPZ sh SCR (constitutive) were transfected on HEK293T cells to generate lentiviral supernatant to infect 6647 cell line. Cells were selected with puromycin (500 ng/ml). For silencing induction cells were exposed to tetracycline 2.5μg/ml.

### Treatment with conventional drugs

Sensitivity to chemotherapeutics was assessed after 72h of treatment with TACS^®^ MTT Cell Proliferation Assay kit (TREVIGEN, Inc.) accordingly to manufacturer's instructions.

### 0662mAb and inhibitors treatment

The anti-CD99 0662 monoclonal antibody (mAb) was produced in the Unité INSERM 343, Hospital de l'Archet, Nice, France. Treatments with 0662mAb (3μg/ml) were performed in adhesion or at the concentration of 5 × 10^6^/ml cells as previously described [20, 21]. After the indicated time-points, samples were tested for Annexin-V/propidium iodide (Mebcyto apoptosis Kit, MBL), protein extraction or specific assays. The following inhibitors were used alone or in combination with 0662mAb: necrostatin-1 (50μM), 3-methyladenine (3-MA) (5mM, Sigma), Cytochalasin D (5μg/ml, Sigma) pre-treatment at 37°C for 60 min, chlorpromazine (10μg/ml, Sigma) pretreatment at 37°C for 30 min, anti-IGF-1R neutralizing antibody AVE1642 [55] (100ng/ml, kindly provided by ImmunoGen Inc), tyrosine kinase inhibitor NVP-AEW541 (1μM, kindly provided by Novartis) pre-treatment at 37°C for 60 min. The Rac1 inhibitor, EHT 1864 (Tocris), was added at 25μM in culture medium 24h after cell seeding and renewed every day. After 48h of treatment, cells were trypsinized, treated with 3μg/ml of 0662mAb for 4h and harvested for vital count. 24h 1% FBS-starved cells were treated with IGF-I (50ng/ml) alone or in combination with 0662mAb.

### Electron microscopy analysis

6647 and LAP-35 untreated or treated cells were fixed with 2.5% glutaraldehyde in 0.1M cacodylate buffer, pH 7.4, post-fixed with 1% OsO4, dehydrated to absolute ethanol and embedded in Epon. Ultrathin sections were stained with uranyl acetate and lead citrate before observation with a Zeiss EM109 electron microscope. Images were captured using a Nikon digital camera Dmx1200F and ACT-1 software.

### Flow cytometry and immunostaining

Standard protocols were followed as described in Supplementary Materials and Methods). For colocalization investigations by immunofluorescence images were acquired and analyzed with a Nikon A1R confocal microscope with a Plan Apo 60x/NA 1.4 DIC N2. Pictures are representative of at least 10 independent fields from three independent experiments. Fields were selected for the presence of cells with the following criteria: well defined limits, clear identification of nucleus and absence of intersection with neighboring cells. Colocalization analysis was performed both with Pearson Colocalization Coefficient (PCC) and Mander's Colocalization Coefficient (MCC, shown in the paper) and calculated by Nis Elements AR4.20.01 software (Nikon). Colocalization index was represented by histograms as mean ± SEM.

### Western blotting and immunoprecipitation

Details about western blotting and immunoprecipitation are provided in Supplementary Materials and Methods.

### CD99 internalization by ELISA and flow cytometry analysis

ELISA assays were performed in 6647 cell line as previously described [56] The expression of CD99 was analyzed by flow cytometry analysis (FACS-Calibur, Becton Dickinson), see Supplementary Materials and Methods.

### Gene expression profile and network analyses

Cell lines were profiled by Agilent-012097 Human 1A Microarray (V2) G4110B 20K arrays. Microarray data are available at GEO database with Series accession number GSE36097 [21]. Enrichment analysis was performed by MetaCore in GeneGO (Thomson Reuters, New York, NY, USA) and by DAVID [57] programs. For details refer to Supplementary Materials and Methods.

### Lucifer yellow incorporation assay

In order to visualize macropinosomes upon treatments, cells were incubated with 0.75 mg/ml Lucifer Yellow (LY) fluid-phase dye (Thermo Scientific) in IMDM 10% FBS for 15 min at 37°C. Tracer incorporation was evaluated by flow cytometry analysis (FACS-Calibur, Becton Dickinson) or by visual imaging (Nikon ECLIPSE 90i with Plan Apo 60x/NA 1.4 DIC N2). Images were captured under identical conditions using a digital color camera (Nikon DS5MC) and the software NIS-Elements AR 3.10 (Nikon).

### Acridine orange immunofluorescence

To track acidic vescicles formation, Acridine Orange (AO, Sigma) staining was performed as described in Supplementary Materials and Methods. Cells were acquired with Nikon ECLIPSE 90i with Plan Fluor 40x/0.75 DIC M/N2 using either a blue light excitation (492 nm) with a 540-550 nm emission filter (lysosomes appear yellowish green), or green light excitation (540 nm) with a long pass >640 nm barrier filter (lysosomes appear red). Images were captured under identical conditions using a digital color camera (Nikon DS5MC) and merged by the software NIS-Elements AR 3.10 (Nikon).

### Statistical analysis

Differences among means were analyzed by Student's *t* test. Fisher's exact test was used to evaluate the statistical association between two variables.

## SUPPLEMENTARY DATA




