# Supplementary material for: CD99 triggering induces methuosis of Ewing sarcoma cells through IGF-1R/RAS/Rac1 signaling
Source: Oncotarget. 2016 Nov 7;7(48):79925–42. doi: 10.18632/oncotarget.13160 (PMC5346761; doi:10.18632/oncotarget.13160)
Supplement: Supplementary file 1 [file oncotarget-07-79925-s001.pdf]

CD99 triggering induces methuosis of Ewing sarcoma cells through IGF-1R/RAS/Rac1 signaling

SUPPLEMENTARY DATA

SUPPLEMENTARY MATERIALS AND METHODS

Plasmids

pLKO.1 shAtg7-E8 (TET-ON inducible) and pGIPZ shSCR (constitutive) were kindly provided by Dr Calabretta, Philadelphia, USA.

pBABE-HRASN17 was kindly provided by Dr Kurosaki, Moriguchi, Japan.

shRNA specific for IGF-1R was cloned into pSilencer™ -2.1.U6 neo (Ambion) plasmid accordingly to manufacturer's instruction. Insert sequence: 5' GATCC GGATTGAGAAAAATGCTGATTCAAGAGATCAGCA TTTTTCTCAATCCTTTTTTGGAAA

3'GCCTAACTCTTTTTACGACTAAGTTCTCTA GTCGTAAAAAGAGTTAGGAAAAAACCTTTTCGA

pCMV-MDM2 was kindly provided by Dr Moretti F, Rome, Italy.

Gene expression analysis

Total RNA was extracted by the TRIzol extraction kit (Life Technologies); quality and quantity were assessed with NanoDrop analysis (NanoDrop Technologies).

Total RNA (500 ng) for each sample was reverse transcribed to cDNA in a 50μl reaction mixture using the High Capacity cDNA Reverse Transcription kit (Applied Biosystems) according to manufacturer's indications. Quantitative real-time PCR was performed on ViiA7 (Life Technologies) using TaqMan PCR Master Mix (Life Technologies).

Predesigned TaqMan probe was used for the target gene IGF-1R (Hs00609566_m1). The relative quantification analysis was performed on the basis of the ΔΔCT method and expression levels of IGF-1R were normalized to that of GAPDH (Hs99999905_m1, Applied Biosystems).

Immunofluorescence staining

EWS cells were seeded on fibronectin-coated coverslips (Sigma) and stimulated after 24h with anti-CD99 0662mAb (3μg/ml). After washing in PBS, cells were fixed in 4% paraformaldehyde and permeabilized in Triton X-100 0.15%-PBS, blocked in 4% BSA and incubated with the following specific antibodies: anti-CD99 primary antibody (Abcam, clone 12E7); anti-RAS (Santa Cruz Biotechnologies, FL-189) anti-LAMP-1 PE (BD Pharmigen); anti-RAB11 (Abcam); anti-clathrin polyclonal antibody (Cell Signaling); anti-caveolin-1 (BD-transduction labs); anti-IGF-1R (Santa Cruz Biotechnologies, clone C20); anti-IGF-1R PE (Pharmigen); anti-RAB5 (Thermo Scientific); anti-Rac1 (Santa Cruz Biotechnologies, clone C14). Nuclei were counterstained with Hoechst 33258.

Immunoistochemistry staining

Sections (5 μm) from formalin-fixed, paraffin-embedded tumor xenografts were placed on poly-L-lysine coated slides (Sigma). Avidin-biotin-peroxidase procedure was used for immunostaining. Briefly, sections were treated sequentially with xylene and ethanol to remove paraffin. For immunohistochemical detection of IGF-IR, sections were pretreated with a citrate buffer solution [0.01 mol/L citric acid and 0.01 mol/L sodium citrate (pH 6.0)] in a microwave oven at 750 W for three cycles of 5 min each. Endogenous peroxidase activity was blocked by treatment with 3% hydrogen peroxide in methanol for 30 min at room temperature. A blocking step with normal goat serum (Vector, Burlinga me, CA) was used. This treatment ensured antigen retrieval from samples. The following primary antibodies (anti-IGF-1R β-subunit, clone C-20, diluted 1:50; anti-RAS, clone FL-189, diluted 1: 100 and anti-Rac1, clone C14, diluted 1: 100, Santa Cruz Biotechnologies) were applied overnight in a moist chamber at 4°C. The following day, tissue sections were incubated with a secondary biotinylated anti-rabbit antibody and with an avidin-biotin-peroxidase complex (Vector).

The final reaction product was revealed by exposure to 0.03% diaminobenzidine (Sigma), and nuclei were counterstained with Mayer's hematoxylin.

Acridine orange (AO) immunofluorescence

6647 and LAP-35 cells were seeded on fibronectin coated-dishes and after 48h stimulated with anti-CD99 0662mAb (3μg/ml) for 3h. After treatment, cells were washed with PBS and then stained with 1 μg/ml AO in PBS for 15 min at 37°C and nuclei were counterstained with vital Hoechst 33342 (Sigma). Apoptosis was induced in 6647 or LAP-35 dominant negative RAS cells (hRAS N17) by treating them with 3μg/ml 0662mAb for 3h at 37°C. Control or treated cells were stained with AO 15 min at 37°C and nuclei were counterstained with vital Hoechst 33342 (Sigma).

Western blotting and immunoprecipitation

Cells were lysed with phospho-protein extraction Buffer (Upstate) supplemented with protease-phosphatase cocktail inhibitor (Sigma). Proteins of interest were detected with specific antibodies: anti-GAPDH (Santa Cruz Biotechnology, FL-335), anti-pan RAS (Thermo Scientific, Ras10), anti-ATG7 (Cell signaling Technologies, 2631), anti-IGF-1R (Santa Cruz Biotechnology, C-20), anti-CD99 (Santa Cruz Biotechnology, 12E7), anti-LC3 (Cell Signaling, D11), phospho-ERK/Thr202-Tyr204 (Covance), anti Rac1 (Santa Cruz Biotechnology, C-14), anti-MDM2 (Calbiochem, 2A10). For immunoprecipitation, 900 µg of cell lysates were incubated for 16h with Protein A/G-Plus-agarose beads (Calbiochem) in the presence of 2 µg of anti-IGF-1R antibody (Calbiochem, αIR-3). Immunoprecipitates and 40 µg total lysates were then resolved on a 10% Tris-HCl gel and immunoblotted with specific antibodies.

CD99 internalization

ELISA assay was performed on 70,000 cells/time-point seeded in multi 24 plates and starved for 3h, then mAb; subsequently cells were washed in TBS 1X, fixed in 3.7% TBS-paraformaldehyde and blocked in 1% TBS-BSA. After the incubation with the anti-mouse secondary antibody, the relative substrate (BIO-RAD Cat# 172-1063) was added to the cells. The reaction was stopped by adding NaOH 0.4 N, then absorbance was read at 405 nm.

CD99 exposure was evaluated by indirect immunofluorescence: after 0662mAb exposure, a pellet of 500 000 cells has incubated with goat anti-mouse IgG (H+L) FITC (Thermo Fisher Scientific Inc) for 30 min, the reaction was stopped by adding serum-free medium. Cells resuspended in 1µg/ml ethidium bromide-PBS were processed for FACS analysis.

Treatment with inhibitor or siRNA of Rac1

The Rac1 inhibitor, EHT 1864 (Tocris), was added at 25 µM in culture medium 24h after cell seeding and renewed every day. After 48h control or treated cells were exposed to 0662mAb and subsequently analyzed. Transient Rac1 depletion was achieved by siRNA approaches using anti-Rac1 oligos commercially available. Scrambled (ON-TARGETplus siCONTROL, Thermo Scientific) was used as control. All were administered at 100 picomoles. Cells were seeded in 60-mm plates in standard medium (3×10^5 cells per plate) and transfected after 24h (Lipofectamine 2000, Life Technologies). 48h later, cells were treated with 3 µg/ml of 0662mAb for 2h and harvested for vital count.

Network analyses of gene expression array

Biological pathways were defined according to “GeneGO Pathway Maps” manually curated database, instead ontologies according to Gene Ontology database. Enrichment was tested for significance by modified Fisher's Exact Test corrected by FDR and considered significant if p-value ≤ 0.1. Network analysis of biologically related terms was performed with the direct interaction method in GeneGO, were an edge connecting two genes indicates their direct biological relation according to GeneGO database.

In vivo treatments with anti-CD99 antibody

Female athymic 4–5-week-old Crl:CD-1nu/nuBR mice (Charles River Italia, Italy) were subcutaneously injected with 5×10^6 EWS cells. The animals were randomised into two groups. In the group treated with anti-CD99 antibody alone, each mouse received s.c. injections of antibody (1mg/injection) in the proximity of the tumor for each day for two subsequent cycles of five days. Control mice received s.c. injections of PBS (same volume/injection; 10 injections). Tumor growth and body weight was assessed once a week by measuring tumor volume, calculated as π/2 [√(a b)]3/6 where a and b are the two maximum diameters. For ethical reasons, mice with local tumors were killed when they achieved a tumor volume of 3 ml and necropsied. All animal experiments were performed according to Italian law 116/92 and European directive 2010/63/UE. Experimental protocols were reviewed and approved by the Institutional Animal Care and Use Committee (“Comitato Etico Scientifico per la Sperimentazione Animale”) of the University of Bologna, and forwarded to the Italian Ministry of Health with letter 4783-X/10 (Responsible Researcher Prof. C. De Giovanni).

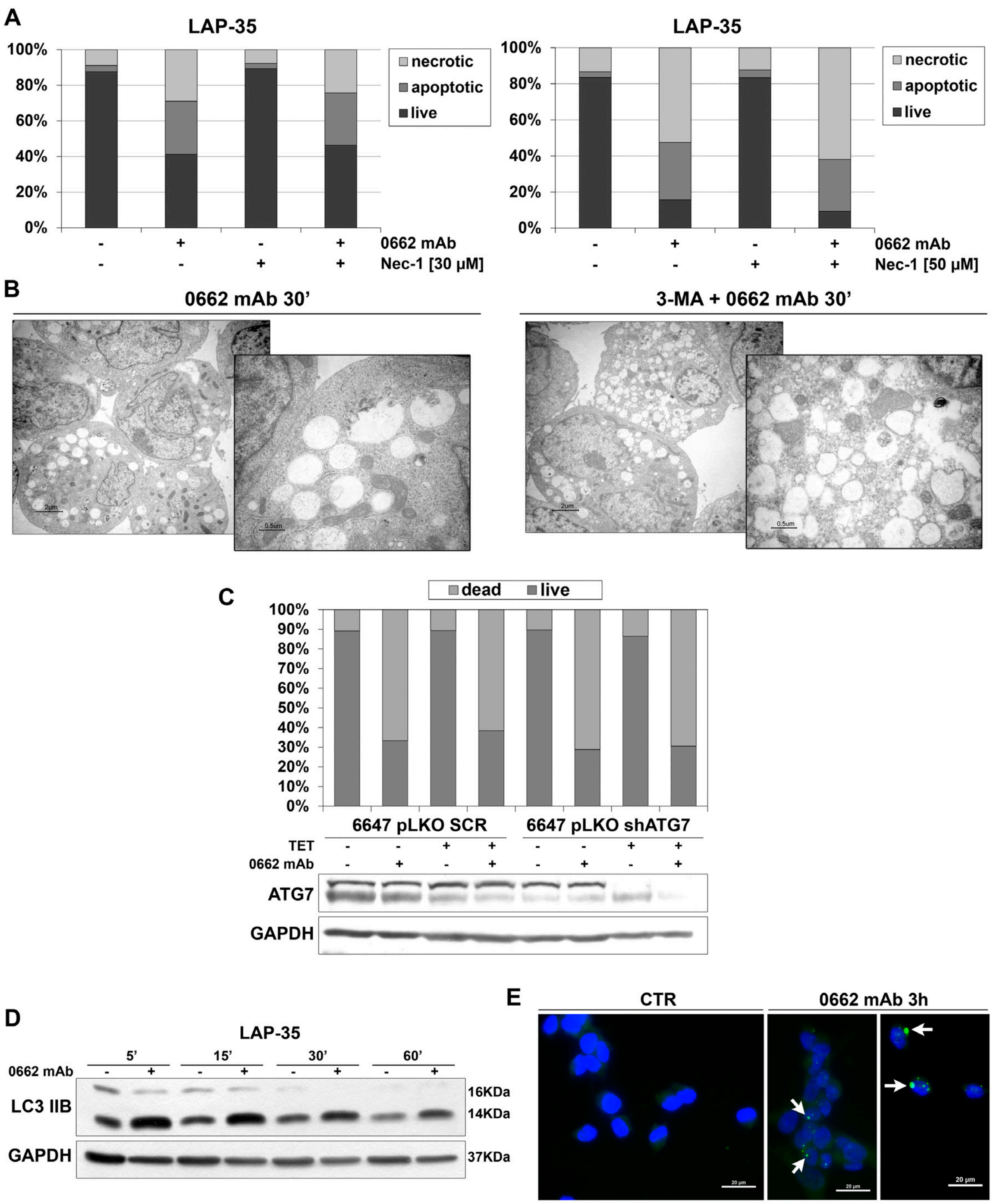

Supplementary Figure S1: Vesicles are induced upon 0662 mAb exposure, but cell death does not occur through necroptosis and it is independent from autophagy. A. Treatment with 0662 mAb (30 min) with or without Nec-1 pretreatment (30μM for 30 min or 50μM overnight) (Annexin V/PI assay). **B.** Transmission electron microscopy of 0662 mAb treated LAP-35 cells with or without 3-MA treatment at low (left, scale bar 2μm) and high magnification (right, scale bar 0.5μm). **C.** Upper panel: percentage of live (dark grey) or dead (propidium iodide-positive; light grey) EWS cells transfected with an inducible system for ATG7 silencing before (-) and after (+) treatment with 0662 mAb. Lower panel: western blotting of ATG7 protein levels before and after treatment with tetracycline (TET) 2.5μg/ml and/or with 0662 3μg/ml in 6647 cells. Equal loading was monitored by GAPDH. **D.** Western blotting of LC3IIB on cell extracts from control (-) or treated LAP-35 cells. Equal loading was monitored by GAPDH. **E.** Immunofluorescence analysis of LC3 in untreated or 0662 mAb treated (3h) LAP-35 cells (scale bar 20μm).

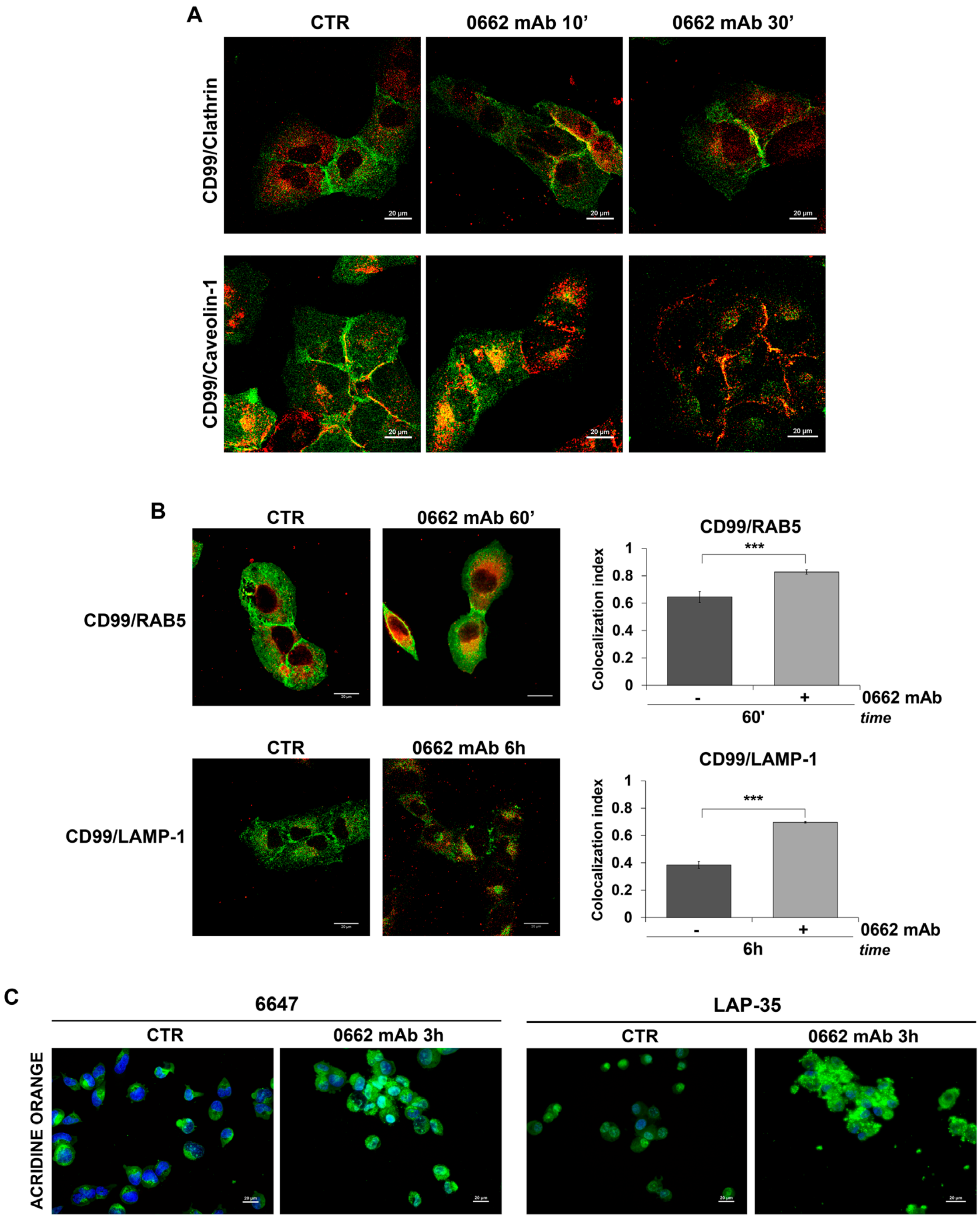

Supplementary Figure S2: A. CD99 internalization after 0662 mAb exposure in U2/CD99wt57 cells. Endogenous colocalization of CD99 and Clathrin (upper panels) or CD99 and Caveolin-1 (bottom panels) in U2/CD99wt57 before (CTR) and after 0662 mAb treatment at the indicated time points. CD99 is labeled in green, Clathrin or Caveolin-1 in red (scale bar 20µm). **B, C.** CD99 is sorted into early endosomes and directed to defective lysosomes. B. Confocal microscopy shows CD99 and RAB5 or CD99 and LAMP-1 colocalization in U2/CD99wt57 cells before and after 0662 mAb treatment at the indicated times. Representative images of CD99 (green) and RAB5 or LAMP-1 (red) staining (scale bar 20µm). Colocalization index (MCC) represented by histograms was calculated by Nis Elements AR4.20.01 (Nikon) as mean ± SEM of an average of one hundred cells from at least 10 independent fields (***p<0.001, Student's t test). C. AO in EWS cells treated with 0662 mAb. Cells were acquired using the microscope Nikon ECLIPSE 90i, Plan Fluor 40X, NA 0.75, DIC M/N2. Pictures provided in the figures are all merged images.

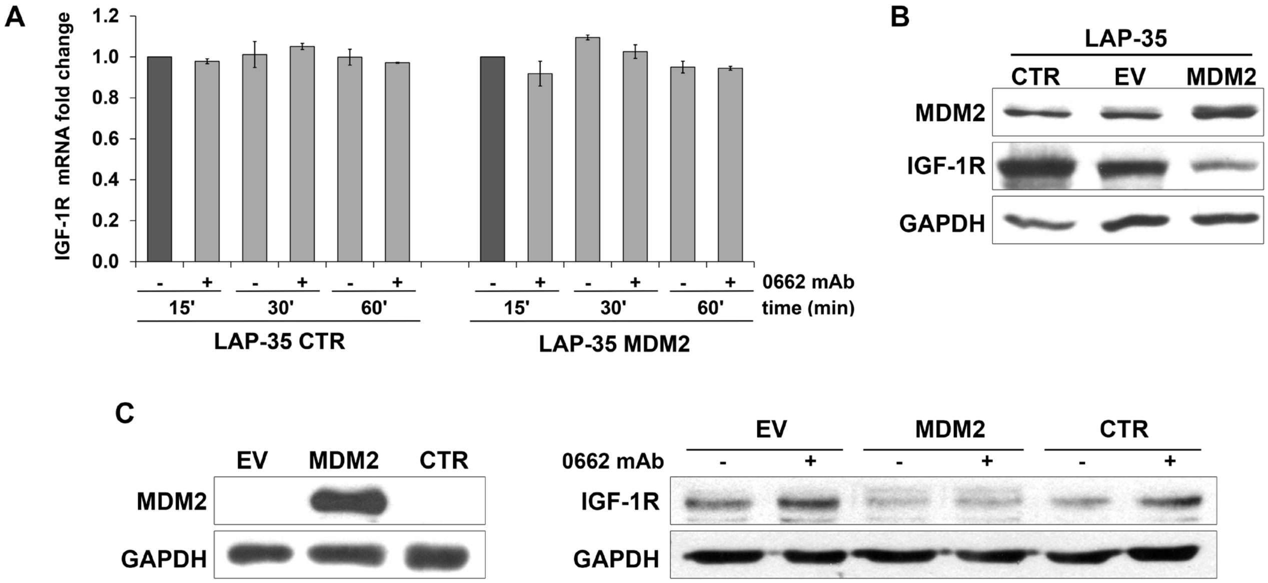

Supplementary Figure S3: Increased levels of IGF-1R are due to reduced degradation mediated by MDM2. A. Histogram shows qRT-PCR evaluation of IGF-1R mRNA levels in LAP-35 or LAP-35-MDM2 expressing cells before (-) and after (+) 0662 mAb treatment. Values are mean ± SEM. GAPDH was used as internal control. **B.** Western blotting of MDM2 and IGF-1R protein levels in corresponding cells. Equal loading was monitored by GAPDH protein levels. **C.** Left panel: western blotting of MDM2 levels in LAP-35 cells (CTR) and LAP-35 cells transfected with empty vector (EV) or plasmid for MDM2 expression (MDM2). Cell lysates were collected after 48 hours of transfection. Equal loading was monitored by GAPDH. Righ panel: western blotting of IGF-1R expression before (-) and after (+) 30 min treatment with 0662 mAb in LAP-35 cells (CTR) and LAP-35 cells transfected for 48 hours with empty vector (EV) or plasmid for MDM2 expression (MDM2). Equal loading was monitored by GAPDH.

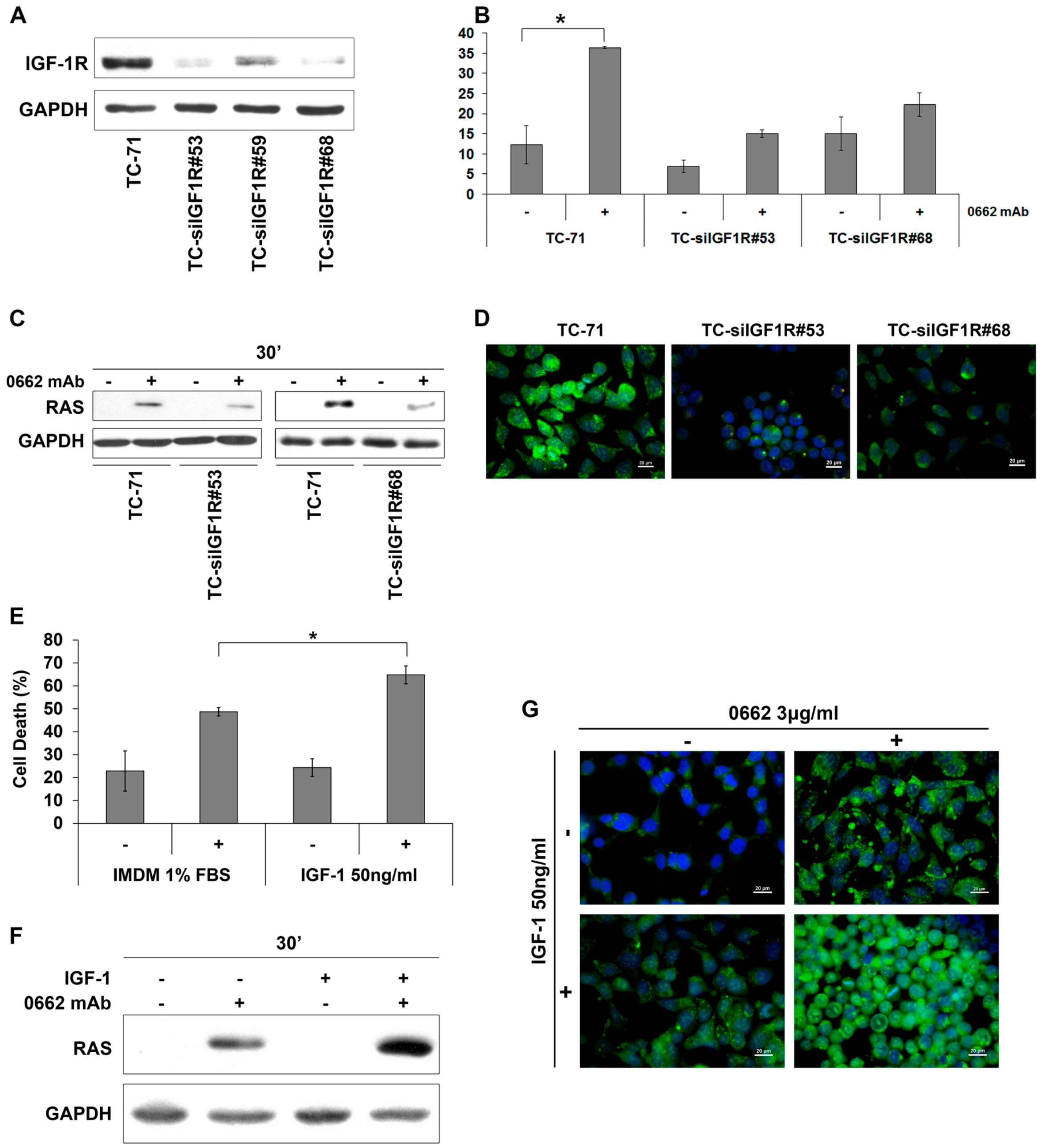

Supplementary Figure S4: CD99-induced cell-death, vacuolization and RAS increase are reverted by IGF-1R silencing or induced by IGF-1 stimulation. A. Western blotting evaluation of IGF-1R levels in TC-71 parental or IGF-1R silenced cells (TC-siIGF1R#53, TC-siIGF1R#59 and TC-siIGF1R#68). GAPDH protein levels are shown as loading control. **B.** Histograms show percentage of dead cells after 0662 mAb treatment (3µg/ml, 30min) in parental or IGF-1R silenced cells. Values are mean ± SEM (Student's t test: *p< 0.05). **C.** Western blotting of RAS levels in parental (TC-71) or IGF-1R silenced cells, before (-) or after (+) treatment with 0662 mAb. GAPDH protein levels are shown as loading control. **D.** Vacuolization assessed by AO after 0662 mAb treatment (3h) in parental (TC-71) or IGF-1R silenced cells. Pictures provided in the figures are all merged images (scale bar 20µm). **E.** Percentage of cell death in TC-71 cell line upon 30 min treatment with 0662 mAb (3µg/ml) alone or in presence of IGF-1 (50 ng/ml) (Annexin V/PI assay). Values are mean ± SEM of three or more independent experiments (Student's t test: *p< 0.05). **F.** Western blotting evaluation of RAS expression in the previous samples. GAPDH protein levels are shown as normalization control. **G.** Vacuolization evaluation with AO staining of TC-71 cells upon treatment with 0662 mAb alone or in presence of IGF-1. For AO staining (D, G), cells were acquired using the microscope Nikon ECLIPSE 90i, Plan Fluor 40X, NA 0.75, DIC M/N2. Pictures provided in the figures are all merged images (scale bar 20µm).

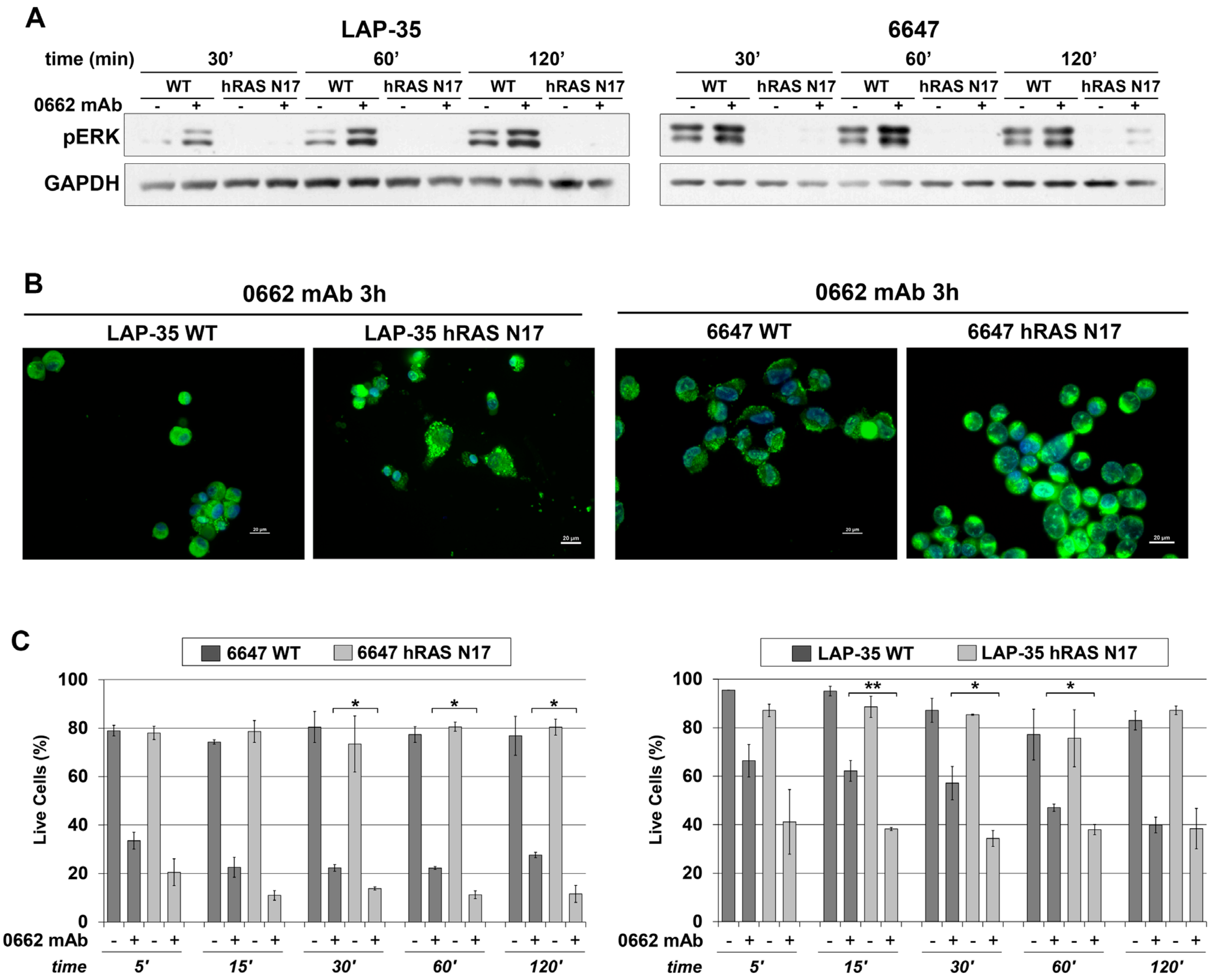

Supplementary Figure S5: CD99-induced cell vacuolization is independent from ERK1/2 activity. A. Western blotting evaluation of phosphorylated p44/p42 ERK1/2 in wild type (WT) or RAS dominant negative (hRAS N17) EWS cells before (-) or after (+) 0662 mAb treatment. GAPDH protein levels are shown as loading control. **B.** AO staining in WT and hRAS N17 EWS cells treated with anti-CD99 0662 mAb. Cells were acquired using the microscope Nikon ECLIPSE 90i, Plan Fluor 40X/0.75 DIC M/N2. Pictures provided in the figures are all merged images (scale bar 20µm). **C.** Histograms show percentage of live cells before (-) and after (+) treatment with 0662 mAb in 6647 and 6647 hRAS N17 (left) or LAP-35 and LAP-35 hRAS N17 (right) (Annexin V/PI assay). Values are mean ± SEM of three or more independent experiments (Student’s t test: *$p < 0.05$; **$p < 0.01$).

Supplementary Table S1: Drug sensitivity compared to CD99 expression and p53 status in EWS cell lines

See Supplementary File 1

Supplementary Table S2: Kegg enriched pathways after 30, 60 and 120 minutes of 0662 mAb treatment

See Supplementary File 1
