# Supplementary material for: CD99 triggering induces methuosis of Ewing sarcoma cells through IGF-1R/RAS/Rac1 signaling
Source: Oncotarget. 2016 Nov 7;7(48):79925–42. doi: 10.18632/oncotarget.13160 (PMC5346761; doi:10.18632/oncotarget.13160)
Supplement: Supplementary file 2 [file oncotarget-07-79925-s002.doc]

**Supplemental Table S1.** Drug sensitivity compared to CD99 expression and p53 status in EWS cell lines

|  | **DRUGS SENSITIVITY** | | | | | **0662**  **SENSITIVITY** | | **CD99 expression** | | **p53 status** |
| --- | --- | --- | --- | --- | --- | --- | --- | --- | --- | --- |
|  | **IC50**  **DX (ng/ml)** | **IC50**  **VCR (ng/ml)** | **IC5**  **IFO (ng/ml)** | **IC50**  **ETO (ng/ml)** | **IC50**  **ACTID (ng/ml)** |  |  | **% positive cells** | **Log Mean Intensity** |  |
|  |  |  |  |  |  | **LIVE** | **DEAD** |  |  |  |
|  |  |  |  |  |  |  |  |  |  |  |
| **LAP-35 CTR** | 3,55  ±0,15 | 1,19  ±0,21 | 3953,7  ±411,84 | 489,65  ±3,35 | 4,94  ±0,58 | 84,7  ±4,7 | 15,3  ±4,7 | 96,9 | 70,5 | WT |
| **+ 0662 mAb** |  |  |  |  |  | 33,3  ±4,6 | **66,7**  ±4,6 |  |  |  |
| **6647 CTR** | 6,6  ±1,0 | 1.6  ±1,2 | 6084  ±2203,85 | 178,07  ±9,36 | 1,98  ±0,98 | 84,8  ± 2,6 | 15,2  ± 2,6 | 98,1 | 113,7 | Point mutation  (p.Ser241Phe) |
| **+ 0662 mAb** |  |  |  |  |  | 17,0  ±3,6 | **83,0**  ±3,6 |  |  |  |
| **RD-ES CTR** | 48.78  ±2,6 | 0,54  ±0.15 | 10475  ±4135 | 219  ±11 | 1.56  ±0.16 | 83,8  ±0,9 | 16,2  ±0,9 | 98,7 | 98,6 | Point mutation  (p.Arg273Cys) |
| **+ 0662 mAb** |  |  |  |  |  | 37,7  ±8,4 | **62,3**  ±8,4 |  |  |  |
| **SK-ES-1 CTR** | 7,2  ±1,2 | 1  ±0,5 | 2920,85  ±1077,35 | 76,22  ±43,88 | 1,95  ±0,41 | 76,3  ±1,2 | 23,7  ±1,2 | 99,8 | 87,5 | Point mutation  (p.Cys176Phe) |
| **+ 0662 mAb** |  |  |  |  |  | 59,4  ±2,4 | **40,4**  ±2,3 |  |  |  |
| **SK-N-MC CTR** | 4,5  ±0,5 | 0,13  ±0,02 | 1466,43  ±233,83 | 252,00  ±10,70 | 1,22  ±0,15 | 86,5  ±1,9 | 13,5  ±1,9 | 98,4 | 68,5 | Major mutation  (c.170_572del) |
| **+ 0662 mAb** |  |  |  |  |  | 75,0  ±0,8 | **25,0**  ±0,8 |  |  |  |
| **H825 CTR** | 46,04  ±4,37 | 7,33  ±0,04 | 1820  ±452,66 | 574  ±215 | 9.85  ±7.47 | 74,4  ±0,2 | 25,6  ±0,2 | 99,1 | 80,2 | Silent mutation  (wt p.arg213arg) |
| **+ 0662 mAb** |  |  |  |  |  | 56,6  ±2,4 | **43,4**  ±2,4 |  |  |  |
| **H-1474-P2 CTR** | 16,25  ±0,63 | 1,48  ±0,78 | 3943,81  ±2784,21 | 182,00  ±61,76 | 0,42  ±0,01 | 74,2  ±1,7 | 25,8  ±1,6 | 99 | 65,1 | WT |
| **+ 0662 mAb** |  |  |  |  |  | 60,3  ±2,1 | **39,7**  ±2,2 |  |  |  |
| **IOR/BRZ_2010 CTR** | 121,74  ±24,04 | 12,79  ±2,81 | 7158,00  ±2033,60 | 268,36  ±31,25 | 10,02  ±2,37 | 78,35  ±1,54 | 21,65  ±1,54 | 97,5 | 93,7 | WT |
| **+ 0662 mAb** |  |  |  |  |  | 54,54  ±0,95 | **45,47**  ±0,94 |  |  |  |
| **TC-71 CTR** | 26,20  ±6,40 | 0,65  ±0,02 | 3711,8  ±918,81 | 279,3  ±14,98 | 0,71  ±0,04 | 80,5  ±2,0 | 19,5  ±2,0 | 99,8 | 75,1 | Major mutation  (p.Arg213X) |
| **+ 0662 mAb** |  |  |  |  |  | 59,4  ±1,4 | **40,6**  ±1,4 |  |  |  |
| **TC/DOXO 8 CTR** | 703  ±116 | 189  ±20 | 7785  ±1585 | 1335  ±429 | 44,25  ±7,25 | 83,51  ±3,05 | 16,49  ±3,05 | 97,3 | 60,01 | Major mutation  (p.Arg213X) |
| **+ 0662 mAb** |  |  |  |  |  | 55,40  ±1,01 | **44,60**  ±1,01 |  |  |  |
| **TC/DOXO 41 CTR** | 424  ±16 | 247  ±73 | 16090  ±2470 | 1256  ±86 | 27,15  ±4,15 | 86,49  ±0,37 | 13,52  ±0,36 | 95,9 | 73,1 | Major mutation  (p.Arg213X) |
| **+ 0662 mAb** |  |  |  |  |  | 49,57  ±1,34 | **50,44**  ±1,33 |  |  |  |
| **TC ET 12nM CTR** | 13855  ±3845 | 1767  ±69 | 8210  ±1990 | 8835  ±665 | 634,15  ± 50,85 | 83,89  ±3,31 | 16,11  ±3,31 | 98 | 89,8 | Major mutation  (p.Arg213X) |
| **+ 0662 mAb** |  |  |  |  |  | 61,26  ±3,39 | **38,75**  ±3,38 |  |  |  |

**Supplemental Table S2**

Kegg enriched pathways after 30, 60 and 120 minutes of 0662 mAb treatment.

| **Term** | **min. p-value** | **30 minutes** | | | **60 minutes** | | | **120 minutes** | | |
| --- | --- | --- | --- | --- | --- | --- | --- | --- | --- | --- |
|  |  | **Count** | **p-value** | **Benjamini** | **Count** | **p-value** | **Benjamini** | **Count** | **p-value** | **Benjamini** |
| hsa04142:Lysosome | 3.36E-07 | 4 | 5.96E-02 | 4.09E-01 | 18 | 3.36E-07 | 4.47E-05 | 11 | 3.65E-04 | 1.16E-02 |
| hsa04512:ECM-receptor interaction | 2.16E-05 |  |  |  | 8 | 2.41E-02 | 3.71E-01 | 11 | 2.16E-05 | 2.07E-03 |
| hsa05200:Pathways in cancer | 5.94E-05 | 7 | 3.69E-02 | 3.63E-01 | 17 | 9.40E-02 | 6.08E-01 | 21 | 5.94E-05 | 2.85E-03 |
| hsa00510:N-Glycan biosynthesis | 8.43E-04 |  |  |  | 8 | 8.43E-04 | 5.45E-02 | 5 | 2.18E-02 | 1.62E-01 |
| hsa00600:Sphingolipid metabolism | 1.88E-03 |  |  |  | 7 | 1.88E-03 | 7.99E-02 | 5 | 1.25E-02 | 1.25E-01 |
| hsa04510:Focal adhesion | 2.40E-03 |  |  |  |  |  |  | 13 | 2.40E-03 | 5.61E-02 |
| hsa00512:O-Glycan biosynthesis | 3.15E-03 |  |  |  | 6 | 3.15E-03 | 9.95E-02 |  |  |  |
| hsa05222:Small cell lung cancer | 3.29E-03 |  |  |  | 7 | 6.67E-02 | 6.01E-01 | 8 | 3.29E-03 | 6.13E-02 |
| hsa04810:Regulation of actin cytoskeleton | 4.18E-03 |  |  |  |  |  |  | 13 | 4.18E-03 | 6.48E-02 |
| hsa00511:Other glycan degradation | 5.60E-03 |  |  |  |  |  |  | 4 | 5.60E-03 | 7.41E-02 |
| hsa04916:Melanogenesis | 6.60E-03 | 5 | 6.60E-03 | 3.28E-01 |  |  |  | 6 | 8.14E-02 | 3.35E-01 |
| hsa05210:Colorectal cancer | 7.60E-03 | 4 | 2.59E-02 | 4.09E-01 | 9 | 7.60E-03 | 1.84E-01 | 7 | 1.33E-02 | 1.21E-01 |
| hsa05217:Basal cell carcinoma | 8.31E-03 | 4 | 8.31E-03 | 2.21E-01 | 6 | 3.88E-02 | 4.82E-01 | 6 | 8.83E-03 | 1.01E-01 |
| hsa00563:Glycosylphosphatidylinositol(GPI)-anchor biosynthesis | 9.54E-03 |  |  |  | 5 | 9.54E-03 | 1.91E-01 |  |  |  |
